# Limb conservation in extremity soft tissue sarcomas with vascular involvement

**DOI:** 10.4103/0019-5413.54969

**Published:** 2009

**Authors:** Rajaraman Ramamurthy, Jagadish Chandra Bose Soundrarajan, Viswanathan Mettupalayam, Subbiah Shanmugham, Balasubramanian Arumugam, Saravanan Periasamy

**Affiliations:** Department of Surgical Oncology, Govt Royapettah Hospital, Chennai, India

**Keywords:** Limb salvage surgery, major vascular infiltration in sarcomas, vessel reconstruction

## Abstract

**Background:**

The major neurovascular involvement and large primary tumors are indication of amputation. The present study is an attempt to explore the feasibility of a limb salvage surgery in extremity sarcoma cases with major vessel involvement. Oncological outcomes and surgery-related morbidities are compared with those reported in literature.

**Materials and Methods:**

A retrospective review of all limb salvage surgeries done in our department between 2005 and 2008 was done and four cases of extremity sarcoma of lower limb involving femoral vessels analyzed. Interpretation of data from these cases, along with review of literature, is done.

**Results:**

In all these cases a wide monobloc excision was done adhering to oncological principles. This required resection of superficial femoral artery alone in two cases, resection of superficial femoral artery along with common femoral vein and femoral nerve in another, and of common femoral vein alone in yet another. Reconstruction was done in all these cases with reversed long saphenous vein graft. Histopathology of resected margins was free of tumor in all the four patients. One patient developed local recurrence and one developed distant metastsis. Two were disease free for one year with good functional limb, one has been disease-free for three years and another was disease-free at two years, after which he defaulted further follow-up. One patient developed arterial blowout which required ligation of common femoral artery which resulted in gangrene of the limb. He underwent amputation.

**Conclusion:**

Major neurovascular involvement in extremity sarcoma is not considered a contraindication for limb salvage surgery. Review of literature also supports our view. Post-operative wound related complications are more in this group of patients. However, long term functional outcome is good. Literature suggests a good long term local control after vascular resection and reconstruction.

## INTRODUCTION

Limb salvage surgery has become the standard of care in managing extremity sarcomas.^1^–^5^ With recent advances in radiotherapy and chemotherapy, 95% of patients with extremity sarcomas will have limb conservation surgery but five per cent of these patients will still require amputation. Common indications for amputation are major neurovascular involvement and large primary tumor where resection would leave a functionally useless limb.

We offered limb salvage to four patients who had major vascular involvement. In this article we share our experience of extremity soft tissue sarcoma in patients, with major vascular involvement, who were successfully treated with limb conservative surgery along with vascular reconstruction.

## MATERIALS AND METHODS

**Figure 1:**
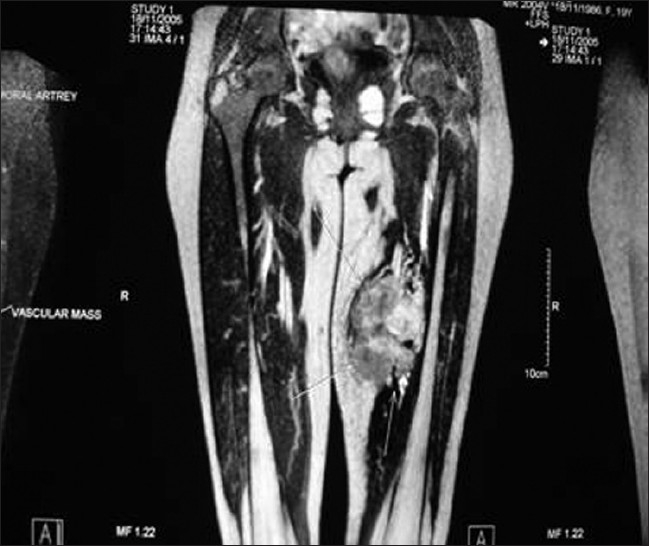
Coronal T2W MRI of patient 1 showing mass involving vessel

**Figure 2:**
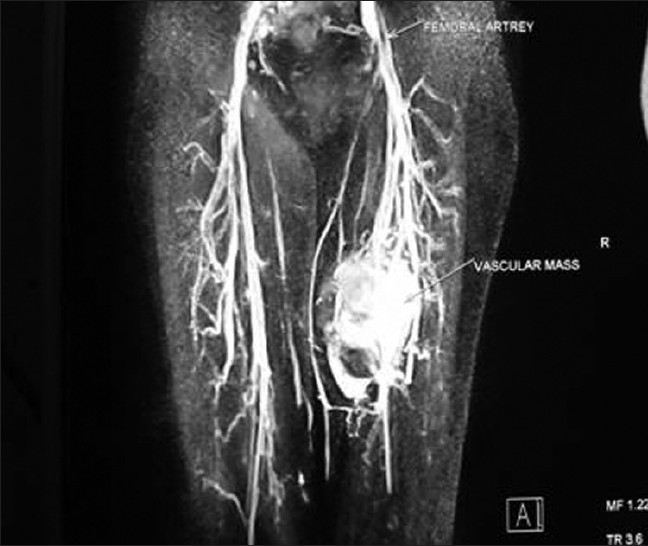
MR angiogram of the same patient showing vessel involvement

A retrospective review of all cases of limb salvage surgery on extremity soft tissue sarcomas cases, in our department, from 2004 to 2008, was done. Four patients who had major vascular involvement diagnosed pre-operatively and offered limb salvage surgery were taken up for analysis. All the four patients presented with complaints of painless swelling in the thigh. Average duration of presenting symptom was four months ranging from three to six months. In three of them vascular involvement was suspected on clinical examination because of weak peripheral pulses. All these patients were subjected to MRI and MR angiogram which confirmed the involvement of major vessel [Figures 1 and 2]. More than 50% circumferential encasement of major vessel in MRI is highly predictive of vascular infiltration by sarcomas. All these patients were subjected to Doppler study which showed loss of triphasic flow and reduced velocity in the involved vessel.

Our routine pre-operative staging workup included chest X-ray, CT scan of the chest and MRI of the involved local part. Histological diagnosis was made by trucut biopsy of the lesion. Biopsy site was chosen along the line of incision. After ruling out distant metastasis all of them were offered limb salvage surgery with curative intent for which they consented.

Surgery was planned according to the imaging extent of the tumor. Along with the muscle group involved a two cm clear margin all around the tumor was marked out in the pre-operative surgical planning sessions. Since vascular involvement was made out pre-operatively, resection and reconstruction of the involved vessel was planned pre-operatively. A wide monobloc excision, along with resection of the involved vessel, was done. Reconstruction of the vessel was done with saphenous vein harvested from opposite leg. All patients were heparinized during the surgery and in the immediate postoperative period. Oral anticoagulation with warfarin was started after five days and heparinization was stopped once the INR reached therapeutic levels. Warfarin was continued for six months.

Follow-up protocol for sarcomas includes clinical examination every month for two years, once in three months for next year, six-monthly up to five years and once yearly after that. Radiological examination with chest X-ray and local part MRI is done once in a year. Follow up protocol for vascular reconstruction group included hand held Doppler study on every visit and color Doppler study once in a year. If patients complain about any symptoms it is evaluated with imaging at any time during the follow-up.

Oncological and functional outcomes of this subset of patients were analyzed. Functional assessment was done with Musculoskeletal Tumor Society rating scale (Enneking score).

## RESULTS

Over a period of four years, between 2005 and 2008, four patients presented to us with extremity soft tissue sarcoma involving the femoral vessels. Demographic details of these patients are given in Table 1. Mean size of the tumor was 13.5 cms. In three of these cases both adductor and anterior compartments of thigh were involved and in one patient only the anterior compartment was involved. Pre-operative diagnosis of vascular involvement was made with MRI and Color Doppler study. In two patients the superficial femoral artery alone was involved. In one patient common femoral vein alone was involved and in one both superficial femoral artery and common femoral vein were involved. Histologies were one each of synovial sarcoma, rhabadomyosarcoma, MPNST and MFH. There were three Grade III sarcomas and one Grade II sarcoma.

**Table 1:** Demographics of soft tissue sarcoma patients with major vessel involvement

	Patient 1	Patient 2	Patient 3	Patient 4
Age	19	63	39	23
Sex	F	M	M	F
Compartment involved	Adductor and anterior compartment of left thigh	Adductor and anterior compartment of right thigh	Anterior compartment of left thigh	Adductor and anterior compartment of left thigh
Size	15 cms	14 cms	10 cms	15 cms
Histology	Synovial sarcoma	Rhabadomyosarcoma	MPNST	MFH
Grade	Gr 2	Gr 3	Gr 3	Gr 3
Vessels involved	Superficial femoral artery	Common femoral vein	Superficial femoral artery	Superficial femoral artery and common femoral vein

**Figure 3:**
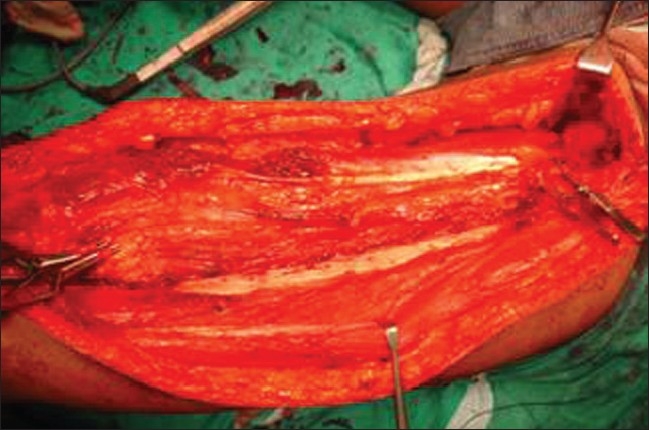
Peroperative photograph showing defect after monobloc excision

**Figure 4:**
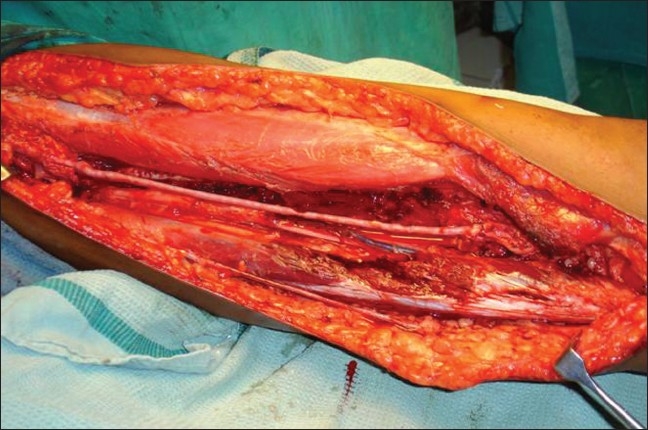
Peroperative photograph showing arterial reconstruction with saphenous graft

**Figure 5:**
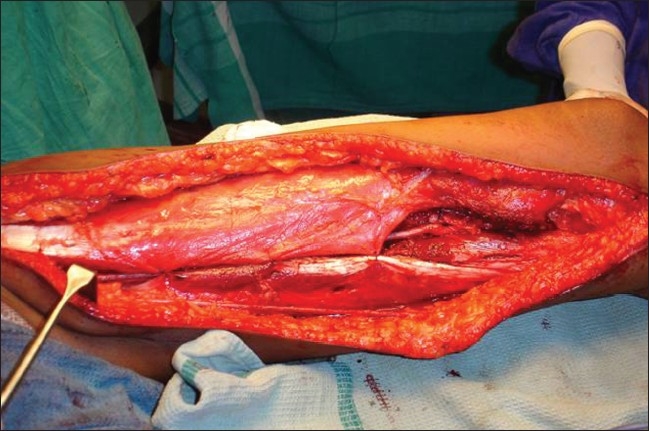
Peroperative photograph showing muscle transposed over vascular reconstruction

Limb salvage surgery in the form of wide monobloc excision was performed on all these patients adhering to oncological principles [Figure 3]. A two cm margin was given all around the tumor and it was excised enbloc with the involved vessel. Two of these patients underwent wide monobloc excision of tumor along with resection of a segment of superficial femoral artery. One underwent wide monobloc excision of tumor along with resection of a segment of superficial femoral artery, common femoral vein and anterior branch of femoral nerve; one had excision of common femoral vein alone with the tumor. Reconstruction was done in all cases with reversed saphanous vein graft harvested from opposite thigh [Figure 4]. An end-to-end anastamosis was done with 6-O prolene. Two patients had only arterial reconstruction while one had both arterial and venous reconstruction. One had venous reconstruction alone [Table 2]. After the vascular reconstruction adjacent muscles were transposed over the reconstructed vessels for protection [Figure 5].

**Table 2:** Operative details of patients who had soft tissue sarcomas involving major vessels

	Patient 1	Patient 2	Patient 3	Patient 4
Vessels resected	SFA	SFA, CFV and anterior branch of FN	SFA and CFV	SFA, CFV and anterior branch of FN
Recontruction	Artery alone	Vein alone	Artery alone	Artery and vein
Graft	Autologus saphenous vein	Autologus saphenous vein	Autologus saphenous vein	Autologus saphenous vein

SFA = Superficial femoral artery, CFV = Common femoral vein, FN = Femoral nerve

Two patients received adjuvant radiation. Adjuvant chemotherapy and radiation was given to one patient.

All the four cases had negative margins on histopathological examination and Vessel infiltration was proved histologically. Three cases were Gr III sarcomas and 1 was Gr II sarcoma. Preoperative histology was confirmed in all cases.

One patient developed in-operable local recurrence and one developed distant recurrence. No patient has developed vascular insufficiency in the follow-up. Three patients reported lower limb edema which was amenable to conservative management (stocking and exercise). Functional assessment was done with Enneking score. Two patients scored 86% (26/30) and 80% (24/30) respectively. One patient scored 70% (21/30) and one patient 40% (12/30).

In the postoperative period one patient developed major flap necrosis and 2 developed minor wound infection. All three patients were managed conservatively. One patient developed vascular blow-out in the immediate post-operative period for which ligation of common femoral artery was done. Later he developed gangrene and underwent amputation [Table 3].

**Table 3:** Outcome of patients who underwent limb salvage surgery for soft tissue sarcomas involving major neurovascular bundle

	Patient 1	Patient 2	Patient 3	Patient 4
Histological margin	Negative	Negative	Negative	Negative
Local or distant recurrence	None	None	None	None
DFS	3 yrs	2 yrs	1 yr	1 yr
Status	Alive NED	Lost to follow-up	Alive with disease	Alive with disease
Functional outcome	Good (86%)	Poor (40%)	Fair (70%)	Good (80%)
Postoperative complication	Minor wound infection	Vascular blowout-requiring amputation	Flap necrosis	Flap necrosis

DFS - Disease free survival

## DISCUSSION

Vascular involvement by soft tissue sarcomas are diagnosed preoperatively with MRI and MR angiogram. MRI is considered the gold standard in diagnosing vascular involvement by soft tissue sarcomas.^6^,^7^ Vascular infiltration by sarcomas necessitating vessel resection is suspected preoperatively if more than 50% of the circumference of the vessel is encased by the tumor. If the tumor is found abutting the neurovascular bundle excision of the tumor along with the peri-advential tissues is sufficient.

Immediate goals in this kind of resection are to achieve a clear histological negative margin and postoperative wound healing with acceptable morbidity. Literature review suggests a high probability of achieving negative surgical margin even in the presence of vascular involvement requiring resection and reconstruction of the involved vessel. Autologus reversed saphenous vein graft is the most commonly used for reconstructing the vessel. In cases where the vein is not available artificial grafts made of poly tetra fluoro ethylene (PTFE) or Dacron can be used.^8^ The incidence of graft related complications are more with artificial grafts.

Frequency of amputation following vascular reconstruction in literature is 15 to 25% which is much higher than limb salvage surgeries without reconstruction (five per cent). Literature reports a higher incidence of wound related complications, as high as 68% after vascular reconstruction.^9^ Explanations given for higher incidence of complications are preoperative radiotherapy and extensive skeletonization of the vessels which lead to de-vascularization of the flaps. Other reported complications are DVT and pulmonary embolism.

Long term goals are to achieve good oncological and functional outcome. Review of the literature reveals a local recurrence rate of 0 to 20 %^10^–^18^ in most large series of limb salvage surgery requiring vascular reconstruction which is similar to the recurrence rate following limb salvage surgery without vascular reconstruction.^9^ Unlike carcinomas, where an increased incidence of distant metastasis is markedly seen with vascular involvement, sarcomas have modest increased incidence of distant metastasis when vessels are involved, 30 vs 10%.^19^

Limb salvage surgery in the presence of neurovascular bundle infiltration necessitating vascular reconstruction is a well established procedure in western literature. Many major centers in India, even today, continue to offer potentially morbid amputations to these patients. Post operative morbidity, fear of oncological outcome and complexity of the procedure associated with vascular reconstruction has deterred many to undertake limb salvage in this subset of patients in India despite being reported in western literature.

In our experience soft tissue sarcomas with vascular involvement are inherently very aggressive tumors which at presentation have large size and high grade. Resection usually involves large amount of tissue resulting in much higher incidence of wound related complication. Reconstruction of the resected vessels is prone to blow outs in the immediate postoperative period and vascular insufficiency in long term. Because of these factors functional outcome can be unpredictable.

Oncologically, sound resection is possible despite the aggressive presentation of these tumors. With addition of radiotherapy good local control can be achieved in good number of these patients. Literature also reports high local control. Although we could not salvage local recurrence in one of our patients, we expect this would not be the case in most local recurrences. These recurrences, in most cases, should be salvageable with amputation. However, distant recurrences would still be a major problem. This should not deter surgeons from performing limb salvage in this subset as these patients still would have a useful limb during their lifetime.

## CONCLUSION

In our experience limb salvage surgery in the presence of major vascular infiltration necessitating vascular reconstruction can be safely performed. Post-operative wound related complications are more in this group of patients. However, long term functional outcome is acceptable. Multi-modality treatment approach is needed in this group of patients to achieve optimal oncological outcome. These aggressive tumors may have poorer outcome but this should not deter surgeons from performing limb salvage in this subset as these patients still would have a useful limb during their lifetime.
